# Physiological and biochemical changes associated with acute experimental dehydration in the desert adapted mouse, *Peromyscus eremicus*

**DOI:** 10.14814/phy2.13218

**Published:** 2017-03-22

**Authors:** Lauren Kordonowy, Kaelina D. Lombardo, Hannah L. Green, Molly D. Dawson, Evice A. Bolton, Sarah LaCourse, Matthew D. MacManes

**Affiliations:** ^1^ Department of Molecular Cellular and Biomedical SciencesUniversity of New HampshireDurhamNew Hampshire; ^2^ Department of Biological SciencesUniversity of New HampshireDurhamNew Hampshire; ^3^ Department of PsychologyUniversity of New HampshireDurhamNew Hampshire

**Keywords:** Dehydration, desert, electrolyte, rodent

## Abstract

Characterizing traits critical for adaptation to a given environment is an important first step in understanding how phenotypes evolve. How animals adapt to the extreme heat and aridity commonplace to deserts is an exceptionally interesting example of these processes, and has been the focus of study for decades. In contrast to those studies, where experiments are conducted on either wild animals or captive animals held in non‐desert conditions, the study described here leverages a unique environmental chamber that replicates desert conditions for captive *Peromyscus eremicus* (cactus mouse). Here, we establish baseline values for daily water intake and for serum electrolytes, as well as the response of these variables to acute experimental dehydration. In brief, *P*. *eremicus* daily water intake is very low. Its serum electrolytes are distinct from many previously studied animals, and its response to acute dehydration is profound, though not suggestive of renal impairment, which is atypical of mammals.

## Introduction

Understanding the evolution of adaptive traits has long been one of the primary goals in evolutionary biology. The study of the relationships between fitness and phenotype, often powered by modern genomic techniques (Vignieri et al. 2010), has provided researchers with insight into the mechanistic processes that underlie adaptive phenotypes (Castoe et al. 2013; Huerta‐Sánchez et al. 2014). Systems in which the power of genomics can be combined with an understanding of natural history and physiology are well suited for the study of adaptation (Mullen et al. 2009; Bedford and Hoekstra 2015), especially when researchers have the ability to assay the link between genotype and phenotype in wild animals and then conduct complementary experiments using representative animals in carefully controlled laboratory environments. The study described here, characterizing the physiology and serum biochemistry of *Peromyscus eremicus* is the first step in a larger study aimed at understanding the genomic architecture of adaptation to desert environments.

Desert adaptation has significant ecological, evolutionary, and biomedical significance. In contrast to humans and other mammals, desert rodents can survive in extreme environmental conditions and are resistant to the effects of dehydration. Physiological adaptions to deserts have been characterized in several rodents. Specifically, renal histology has been studied in multiple Heteromyid rodents (Altschuler et al. 1979), and the general conclusion is that these desert adapted animals have evolved elongate Loops of Henle (Barrett et al. 1978; Mbassa 1988; Beuchat 1996) that are hypothesized to optimize water conservation. In addition to studies of renal histology, several studies have characterized pulmonary water loss (Schmidt‐Nielsen and Schmidt‐Nielsen 1950; Hayes et al. 1998), water metabolism (Howell and Gersh 1935), and water consumption (MacMillen and Lee 1967; Bradford 1974; Mares 1977; Nagy 1988; Merkt and Taylor 1994) in desert rodents. While desert animals possess specialized physiology that is efficient with regard to water metabolism and loss, whether or not specialized genomic adaptation exists is an active area of research (MacManes and Eisen 2014; Marra et al. 2014, 2012).

Although the cactus mouse (*P. eremicus*) has not been a particular focus for the study of desert adaptation (but see Al‐Kahtani et al. 2004; MacManes and Eisen 2014), this Cricetid rodent native to the arid regions of the Southwestern United States and Northern Mexico (Veal and Caire 1979) offers a unique opportunity to understand physiological adaptations to deserts. *P. eremicus* is a member of a larger genus of animals known colloquially as the “*Drosophila of mammals*”, and *Peromyscus* species have been the focus of extensive study (Hoekstra et al. 2001; Steiner et al. 2007; MacManes and Lacey 2012; Shorter et al. 2012). *P. eremicus* is a sister species to the non‐desert adapted *Peromyscus californicus* (Bradley et al. 2007), and it is closely related to *Peromyscus crinitus*, the canyon mouse, another desert adapted rodent native to southwestern deserts.

Critical to desert survival is the ability to maintain water balance when acute loss exceeds dietary intake (Heimeier et al. 2002). Indeed, the mammalian corpus consists of 60% water (Jéquier and Constant 2009). Far from a static reservoir, proper physiologic function requires water for numerous processes, including nutrient transport (Haussinger 1996), signal transduction, pH balance, thermal regulation (Montain et al. 1999) and the removal of metabolic waste. To accomplish these functions, a nearly constant supply of water is required to replace water loss (Jéquier and Constant 2009), which occurs mainly via the gastrointestinal and genitourinary systems, and evaporative loss, which is greatly accelerated in extreme heat and aridity (Cheuvront et al. 2010). Because the body possesses limited water reserves, when loss exceeds intake during even a short period of time, dehydration and death can occur. Mammals are exquisitely sensitive to dehydration and possess limited compensatory mechanisms.

Characterizing desert adaptation requires careful and integrative physiological studies, which should include a detailed characterization of water intake, responses to dehydration, and the measurement of blood electrolytes. Indeed, quantifying these metrics is one of the first steps in understanding how animals survive in the extreme heat and aridity of deserts. In particular, the electrolytes chloride and sodium are important markers of dehydration (Costill et al. 1976). These molecules play essential roles in metabolic and physiological processes, and they are integral to the functionality of a variety of transmembrane transport pumps (Blaustein and Lederer 1999; Jentsch et al. 2002), neurotransmission (Yu and Catterall 2003), and maintenance of tonicity (Feig and McCurdy 1977). Furthermore, hypernatremia causes restlessness, lethargy, muscle weakness, or coma (Adrogué and Madias 2000). Bicarbonate ion, in contrast, is primarily responsible for aiding in the maintenance of acid‐base balance and is resorbed in the renal tubules (McKinney and Burg 1977). Blood urea nitrogen (BUN) assays the abundance of urea – the end‐product for metabolism of nitrogen containing compounds. Urea is resorbed in the glomerulus, and renal impairment is often inferred when BUN becomes elevated (Baum et al. 1975). Importantly, the canonical model of urea resorption is dependent on urine volume, which is markedly diminished in desert rodents, thus limiting the utility of using BUN as an indicator of renal function. Lastly, creatinine, a product of muscle breakdown, whose measured level does not depend on urine volume, is used as a measure of renal function (Baum et al. 1975).

Genes most frequently implicated in desert‐adaptation include members of the aquaporin family (Huang et al. 2001). However, previous work suggests that an alternative gene family, the solute carriers, are more relevant for desert‐adaptation in the cactus mouse (MacManes and Eisen 2014). As a first step toward fully elucidating the patterns of adaptive evolution to deserts in *P. eremicus*, we characterized the normal patterns of water intake and electrolyte levels, as well as the physiologic response to experimental dehydration. As such, this study provides critical physiological and biochemical information about *P. eremicus* and its response to dehydration, and is generally useful as researchers begin to leverage large‐scale genome data against classic questions regarding the evolution of adaptive phenotypes.

## Materials and Methods

We used captive *P. eremicus* (*n* = 44, 24 male, 20 female) that were descendant from mice purchased from the University of South Carolina Peromyscus Genetic Stock Center. The USC colony was founded using wild caught animals from a dry‐desert population in Arizona. For ongoing experimental purposes, animals are housed in a large walk‐in environmental chamber built to replicate the environmental conditions in which this population has evolved. Specifically, the animals experience a normal diurnal pattern of temperature fluctuation, ranging from 90 F during the daytime to 75 F during the night. Relative humidity (RH) ranges from 10% during the day to 25% during the night. Animals are housed in standard lab mouse cages with bedding that has been dehydrated to match desert conditions. They are fed a standard rodent chow, which has also been dehydrated. Water is provided ad libitum during certain phases of experimentation and withheld completely during others. All animal care procedures follow the guidelines established by the American Society of Mammalogy (Sikes and Gannon 2011) and have been approved by the University of New Hampshire Animal Care and Use Committee under protocol number 103092.

All animals included in this study were sexually mature adults, as defined for males as having scrotal testes and for females as having a perforate vaginal meatus. A slight bias for the inclusion of males exists, as a concurrent study of male reproductive genomics was occurring. Preliminary analyses conducted suggest that no significant differences in any of the physiological measures, and thus, males and females were analyzed as one group. For a subset of animals, water intake was measured, which was accomplished via the use of customized 15 mL conical tubes, wherein water intake was measured every 24 h for a minimum of three consecutive days (range 3–10 days). Animals selected for the dehydration trial were weighed on a digital scale, housed without water for 3 days, then reweighed to determine the change in body mass due to dehydration. At the conclusion of water measurement or after three days of acute dehydration, animals were sacrificed via isoflurane overdose and decapitation. Immediately after death, a 120 μL sample of trunk blood was obtained for serum electrolyte measurement. This was accomplished using an Abaxis Vetscan VS2 machine with a critical care cartridge, which measures the concentration of several electrolytes (sodium, chloride, bicarbonate ion, creatinine, and blood urea nitrogen (BUN)) relevant to hydration status and renal function. Lastly, the kidney, spleen, liver, lung, hypothalamus, testes, vas deferens and epididymis were dissected out and stored in RNAlater (Ambion Inc.) for future study. All statistical analyses were conducted in the statistical package R (R Core Development Team, 2011).

## Results

**Figure 1:**
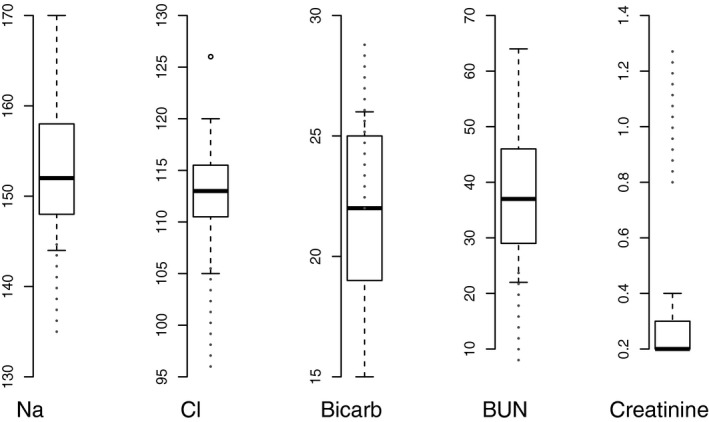
Normal values (*n* = 44, 24 male, 20 female) for serum electrolytes. Human normal values (from Medline) are plotted for comparison in dotted lines. Of note, the Abaxis VS2 electrolyte analyzer does not measure creatinine below 0.2 mg/dL, and therefore the range for normal creatinine is truncated at this value.

We measured the daily water intake for 44 adult cactus mice (24 male, 20 female) for between three and 10 consecutive days. Mean water intake was 0.11 mL per day per gram body weight (median = 0.11, SD = 0.05, min = 0.033, max = 0.23). We measured levels of serum sodium, chloride, bicarbonate ion, creatinine, and blood urea nitrogen (BUN) for the same 44 adult mice, thereby establishing normal (baseline) values for *P. eremicus* (Fig. 1 and Table 1).

**Table 1:** Normal values for serum electrolytes. Normal values (*n* = 44, 24 male, 20 female) are defined as those values falling between the 1^st^ and 3^rd^ quartile

	Normal	Min	Max	Mean
Sodium (mmol/L)	148–158	144	170	153
Chloride (mmol/L)	110–115	105	126	113
BUN (mg/dL)	29–46	22	64	37
Bicarb (mmol/L)	19–25	15	26	22
Creatinine (mg/dL)	>0.2–0.3	>0.2	0.4	0.22

Of note, the Abaxis VS2 electrolyte analyzer does not measure creatinine below 0.2 mg/dL; therefore, the range for normal creatinine is truncated at this value.

**Figure 2:**
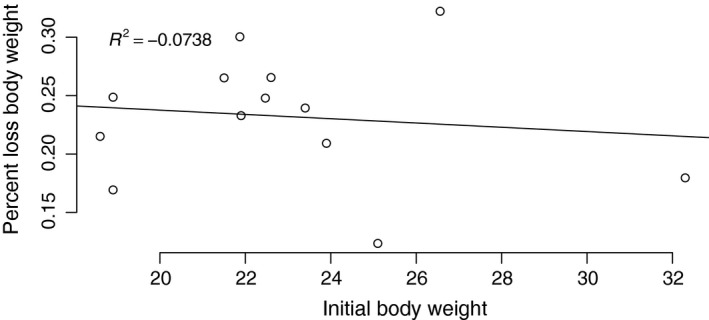
Percent body weight loss as a function of initial body weight due to experimental dehydration. No significant trend exists (*n* = 13, seven males, six females).

A comparison of mice provided with water ad libitum to mice exposed to experimental water deprivation for 3 days revealed that the dehydrated mice lost an average of 23.2% of their body weight (median = 23.9%, SD = 5.3%, min = 12.3%, max = 32.3%, *n* = 13 dehydration treatment, seven males, six females). Despite this substantial weight loss, anecdotally, mice appeared healthy. They were active, eating, and interacting normally with handlers and other mice. The amount of weight loss did not depend on daily water intake (*P* = 0.63, *r* ^2^ = 0.03), though the trend suggests that animals that drank more water lost more weight). Furthermore, initial body weight did not strongly influence the percent loss of body weight due to acute dehydration (Fig. 2; *P* = 0.68, *r* ^2^ = 0.02).

**Figure 3:**
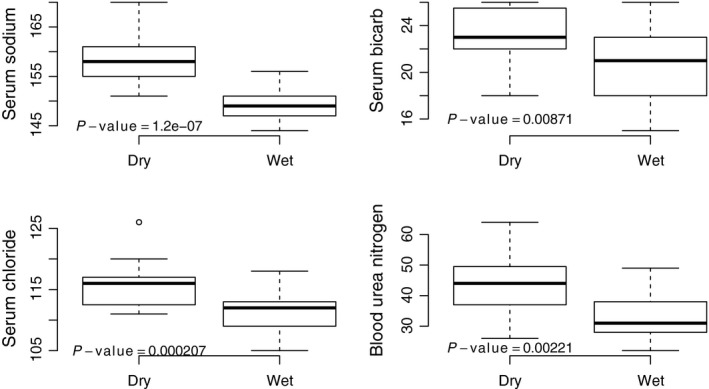
Experimental dehydration resulted in increases in serum sodium, chloride, BUN and bicarbonate ion. Reported *P*‐values are from a two‐tailed t‐test (*n* = 13 dehydrated: DRY,* n* = 31 hydrated: Wet).

In addition to a substantial loss in body weight, dehydration was associated with differences in serum electrolytes (Fig. 3; *n* = 13 dehydrated, *n* = 31 hydrated). These changes were subtle, but significant using a two‐sample t‐test (*P* < 0.008 in all cases).

**Figure 4:**
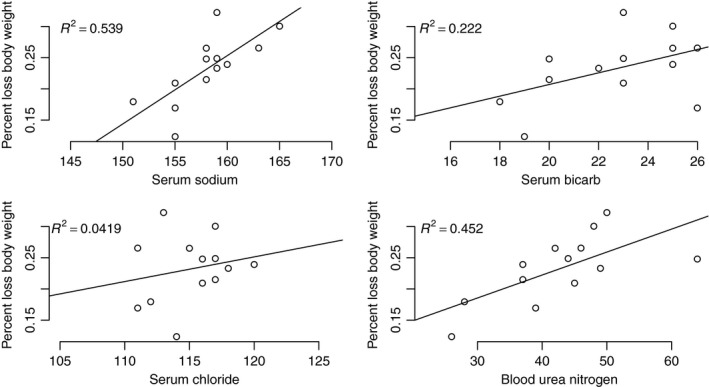
The relationship between serum electrolytes is positive in all cases and significant for sodium (F‐statistic: 12.85, 11 DF,* P*‐value: 0.004283) and BUN (F‐statistic: 9.089, 11 DF,* P*‐value: 0.01177) *n* = 13, seven males, six females.

Lastly, the levels of serum electrolytes were tightly correlated with percent body weight loss (Fig. 4). Indeed, the relationship between the level of serum sodium and weight loss was positive and significant, (ANOVA, F‐statistic: 12.85, 11 DF, *P* = 0.004), as was the relationship between BUN and weight loss (ANOVA, F‐statistic: 9.089, 11 DF, *P* = 0.012). The relationships between weight loss and chloride and bicarbonate levels, respectively, were positive but not significant. Of note, all data are available at https://github.com/macmanes-lab/peer_rnaseq/blob/master/phys4.csv.

## Discussion

Deserts are among the harshest environments on the planet. Indeed, animals living in these areas must be highly adapted to the unique combination of extreme heat and aridity. Given that our understanding of the physiology of desert adapted animals is limited largely to studies in renal histology (Mbassa 1988) and on water intake and output (MacMillen and Lee 1967; Tracy and Walsberg 2001), an enhanced understanding of serum electrolyte changes due to dehydration is informative. Because many of the harmful effects of dehydration result from electrolyte abnormalities, characterizing normal values and the electrolyte response to dehydration represents a critical first step in garnering a deeper understanding of how desert animals survive despite severe and prolonged dehydration.

In this study, normal (baseline) values for serum Sodium, Chloride, Bicarbonate Ion, Creatinine, and blood urea nitrogen were established in a captive colony of lab animals housed in desert conditions. Although these measures may differ in wild animals (see Calisi and Bentley 2009) for a brief review of such differences), establishing normal values in captive animals is crucial, though future studies aim to understand the patterns of electrolyte variation in wild animals. In *P. eremicus*, we define the normal ranges for each electrolyte as those values falling between the 1^st^ and 3^rd^ quartile. Serum chloride and sodium were significantly higher than in published ranges for other mammals, including humans, a marsupial (Viggers and Lindenmayer 1996), *Cricetomys* (Nssien et al. 2002), and the porcupine (Moreau et al. 2003). However, serum chloride and sodium levels in our study were quite comparable to another wild rodent, *Neotoma fuscipes* (Weber et al. 2002), a Mustelid (Thornton et al. 1979), and the Hyrax (Aroch et al. 2007). Values for BUN are generally higher in this study; unfortunately, a direct comparison is not possible, as measured values are dependent on the volume of urine produced. Serum creatinine is low, largely resulting from the general lack of muscle mass in *P. eremicus* relative to other mammals. However, because the equipment used to analyze this electrolyte does not effectively capture the lower end of the biological range, direct comparisons are not made for this metric. Future measurements, using a more sensitive HPLC method for quantitating serum creatinine will improve our ability to detect more subtle changes.

In addition to characterizing baseline electrolytes and their response to experimental dehydration, the normative value for daily water intake was estimated to be 0.11 mL per day per gram body weight. Though comparable measures of water consumption are scarce, one study of two arid adapted *Limoys* (*L. pictus* and *L. irroratus*) housed in non‐desert captive settings were estimated to be 0.18 and 0.17 mL per day per gram body weight respectively (Christian et al. 1978) – a value much greater than in *P. eremicus*.

Animals that were exposed to experimental dehydration lost a substantial amount of body weight. Dehydration in humans, resulting in loss of even a fraction of this amount results in cardiovascular collapse and death (Mehta et al. 2015). Indeed, even a dehydration‐related loss of a few percent of body weight may cause serious renal impairment or renal failure. That the cactus mouse may lose so much weight as a result of dehydration and remain active, and apparently healthy, without renal impairment, is a testament to their desert adaptation. The magnitude of weight loss and the negative (though non‐significant) relationship between baseline weight and weight loss, coupled with the lack of behavioral impairments suggests that metabolic water production via the oxidation of fat may be an important and potentially adaptive mechanism preventing more serious complications from acute dehydration. Indeed, water metabolism may produce a substantial amount of water (reviewed in Johnson et al. 2016), which has been demonstrated in a diverse group of animals including marine mammals (Ortiz 2001) and desert rodents (Frank 1988). Future studies of fat metabolism in *P. eremicus*, using computed tomography, are planned. Because animals are essentially anuric, particularly when dehydrated, direct measurement of fax oxidation (e.g. urine beta hydroxybutryate) is not possible.

As described above, mice appeared to be grossly behaviorally intact. Despite this, they may be experiencing a degree of cognitive impairment, as is the case with human dehydration, where even mild‐dehydration is associated with cognitive impairment (Armstrong et al. 2012). Future studies, using classical Y‐maze and novel object recognition tests aim to understand more fully the cognitive effects of dehydration in cactus mouse.

In addition to weight loss, dehydrated animals demonstrated biochemical evidence of physiological stress, in the form of increased sodium, chloride, BUN, and bicarb. There were no significant relationships between any physiological value and creatinine, suggesting that dehydration related stress does not result in renal impairment or damage. Indeed, this is in contrast to humans and other mammals, where acute dehydration of the nature imposed on these animals is universally related to renal failure and subsequent death. That *P. eremicus* can withstand this level of dehydration is a testament to the processes involved in adaptation.

In summary, we present a set of physiological measurements that represent the endpoints in the physiological management of acute dehydration in the desert adapted cactus mouse. How these endpoints are achieved is an outstanding question deserving of future study, particularly in light of global climate change (Johnson et al. 2016). For instance, Vasopressin, along with the Renin‐Angiotensin‐Aldosterone system are thought to be a critically important to the regulation of water and solute balance (Weaver et al. 1994; Nielsen et al. 1995; Bankir et al. 2005; Zuber et al. 2007; Muñoz et al. 2010). Comparative genomic analysis, studies of gene expression, and the measurement of protein levels will provide important insights into the actual mechanisms underlying these phenotypes.

In addition to understanding the mechanisms of salt and water balance, characterizing the ways in which desert animals prevent dehydration‐linked renal failure is exceptionally important. Unlike in humans, where repeated dehydration events lead to a progressive decline in renal function (Glaser et al. 2016), it is hypothesized that repeated acute‐dehydration is unlikely to be linked to renal failure in animals that have evolved in desert environments. Testing this hypothesis, along with understanding the mechanisms which limit renal damage (e.g., modulating renal microcirculation, maintaining cell volume via organic osmolytes) could provide previously uncharacterized clinically relevant insights into renal (dis) function.

## Conflict of Interest

The authors declare no conflict of interest.
